# Supplementary material for: TRPC5 controls the adrenaline-mediated counter regulation of hypoglycemia
Source: EMBO J. 2024 Oct 7;43(23):5813–36. doi: 10.1038/s44318-024-00231-0 (PMC11612138; doi:10.1038/s44318-024-00231-0)
Supplement: Supplementary file 1 — Appendix [file 44318_2024_231_MOESM1_ESM.pdf]

Appendix to:

TRPC5 controls the adrenaline-mediated counter regulation of hypoglycemia

Jenny Bröker-Lai*[1,2], José Rego Terol*[3], Christin Richter[#1], Ilka Mathar[#1,2], Angela Wirth[#1,2], Stefan Kopf[#4, 10], Ana Moreno-Pérez[3], Michael Büttner[5], Linette Liqi Tan[1], Mazen Makke[3], Gernot Poschet[5], Julia Hermann[1], Volodymyr Tsvilovskyy[1,2], Uwe Haberkorn[6], Philipp Wartenberg[9], Michael Berlin[1,2], Roger Ottenheijm[1,2], Koenraad Philippaert[1,2], Moya Wu[10], Tobias Wiedemann[10], Stephan Herzig[10], Anouar Belkacemi[1,2], Rebecca T Levinson[11], Nitin Agarwal[1], Juan E. Camacho Londoño[1, 2], Bert Klebl[8], Klaus Dinkel[8], Frank Zufall[3], Peter Nussbaumer[8], Ulrich Boehm[7], Rüdiger Hell[5], Peter Nawroth[4, 12], Lutz Birnbaumer[9], Trese Leinders-Zufall[3], Rohini Kuner[1], Markus Zorn[4], Dieter Bruns[3], Yvonne Schwarz[3]* and Marc Freichel[§1,2]*

* shared first and senior authorship #* Contributed equally

§ Contact Info: ***Marc Freichel***

Institute of Pharmacology, Heidelberg University, Heidelberg, Germany
Email: Marc.Freichel@pharma.uni-heidelberg.de

Yvonne Schwarz
Center for Integrative Physiology and Molecular Medicine, Saarland University
Homburg, Germany
Email: Yvonne.Schwarz@uks.eu

1 Institute of Pharmacology, Heidelberg University, Heidelberg, Germany

2 DZHK (German Centre for Cardiovascular Research), partner site Heidelberg/Mannheim, Heidelberg, Germany

3 Center for Integrative Physiology and Molecular Medicine (CIPMM), Saarland University, Homburg, Germany

4 Klinik für Endokrinologie, Diabetologie, Stoffwechsel und Klinische Chemie, Heidelberg, Germany

5 Metabolomics Core Technology Platform, Centre for Organismal Studies Heidelberg (COS Heidelberg), Heidelberg, Germany

6 Nuclear Medicine, Heidelberg University Hospital, Heidelberg, Germany

7 Experimental and Clinical Pharmacology and Toxicology, Center for Molecular Signaling (PZMS), Saarland University, Homburg, Germany

8 Lead Discovery Center GmbH, Dortmund, Germany

9 Institute of Biomedical Research (BIOMED UCA CONICET) Edificio San José, Piso 3 School of Biomedical Sciences, Pontifical Catholic University of Argentina

10 Institute for Diabetes and Cancer, Helmholtz Diabetes Center, Helmholtz Zentrum Muenchen, German Research Center for Environmental Health (GmbH), 85764 Neuherberg, Germany.

11 Department of General Internal Medicine and Psychosomatics, Heidelberg University Hospital, Heidelberg, Germany.

12 Deutsches Zentrum für Diabetesforschung (DZD e.V), München-Neuherberg, Germany

Table of contents

Table of contents …………………………………………………………………………………… 2

Appendix Figure S1: Deletion of the TRPC subtype TRPC5 is sufficient for a defective autonomic counter regulation of insulin-induced hypoglycemia ………………………... 3

Appendix Figure S2: Stress-induced adrenaline secretion in *Trpc5-deficient* mice ……………...4

Appendix Figure S3: The interplay between glucagon and cortisol is not altered in TRPC5 deficient mice ……………………………………………………………………………….. 5

Appendix Figure S4: Analysis of the role of TRPC5 in catecholaminergic neurons of the VLM and NTS………………………………………………………………………………… 6

Appendix Figure S5: Properties of the amperometric release events are not affected by TRPC5 activity ……………………………………………………………………………………… 7

Appendix Figure S6: Genetic loss of TRPC5 does not alter quantal size of catecholamine secretion in response to muscarine stimulation …………………………………… 8

Appendix Figure S7: PACAP-activated cation currents and phospholipase C dependent catecholamine secretion in chromaffin cells from wt and *Trpc5*$^{–/0}$ mice …………………………… 9

Appendix Figure S8: Alterations in plasma amino acids, TCA cycle metabolites and fatty acids in *Trpc5*$^{fx/0}$*;DBH-Cre*$^{+}$ and *Trpc5*$^{fx/0}$*;DBH-Cre*$^{–}$ mice under insulin evoked hypoglycemia ………………………………………………………………………………...10

Appendix Figure S9: Analysis of plasma amino acids, TCA cycle metabolites and fatty acids in HAAF patients under insulin evoked hypoglycemia ……………………………… 12

Appendix Figure S10: Flowchart, inclusion and exclusion criteria for the patients recruited in the study ………………………………………………………………………………...13

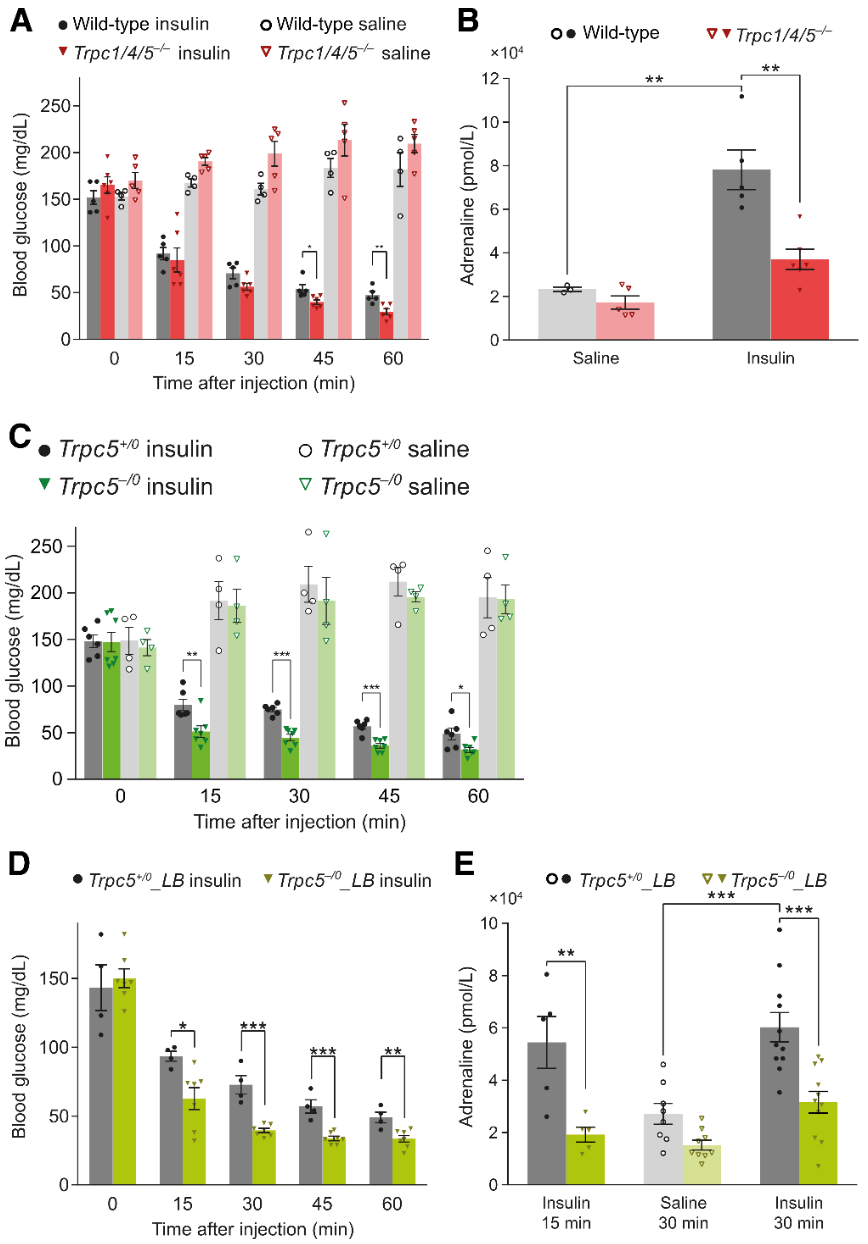

Appendix Figure S1: Deletion of the TRPC subtype TRPC5 is sufficient for a defective autonomic counter regulation of insulin-induced hypoglycemia.

(A) Time course of the blood glucose during an insulin tolerance test (ITT) on *Trpc1/4/5–/–* mice (*Trpc1/4/5–/–*: n = 6 for insulin, n = 5 for saline; wild-type: n = 5 for insulin, n = 4 for saline). Insulin, 45 min p = 0.0185; 60 min, p = 0.00523. **(B)** Plasma adrenalin levels 60 min after insulin injection (*Trpc1/4/5–/–*: n = 6 for insulin, n = 5 for saline; wild-type: p = 0.00212, n = 5 for insulin, n = 3 for salinet; insulin, wild-type vs. *Trpc1/4/5–/–* p = 0.00403). **(C)** Insulin tolerance tests (ITT) on *Trpc5–/0* mice and litter-matched controls (*Trpc5+/0*) arising from matings of *Trpc5+/–* females and *Trpc5–/0* males: Time course of the blood glucose levels after insulin injection is shown. (*Trpc5–/0*: n = 7 for insulin, n = 4 for saline; *Trpc5+/0*: n = 6 for insulin, n = 4 for saline). Insulin, 15 min p = 0.00764; 30 min p = 2.6×10^{-5}; 45 min p = 0.000154; 60 min p = 0.0185. **(D)** Time course of the blood glucose during an ITT on litter-matched *Trpc5–/0_LB mice* (*Trpc5–/0_LB*: n = 7; *Trpc5+/0_LB*: n = 4), p = 0.0213 (15 min), p = 0.000139 (30 min), p = 0.000292 (45 min), p = 0.00579 (60 min). **(E)** Plasma adrenaline levels 15 min and 30 min after injection of insulin in *Trpc5–/0_LB* litter-matched mice *(Trpc5–/0_LB*: n = 5 for insulin (15 min), n = 11 for insulin (30 min), n = 9 for saline; *Trpc5+/0_LB*: n = 5 for insulin (15 min), n = 11 for insulin (30 min), n = 8 for saline). Two-way ANOVA, p = 3.01×10^{-8}, with Bonferroni pairwise comparison, p = 0.00442 (15 min), p = 3.38×10^{-4} (30 min, insulin) and p = 1.36×10^{-4} (*Trpc5+/0_LB*, 30 min, saline vs. insulin). (A-E) Mean ± s.e.m., * p <0.05; ** p <0.01; *** p<0.001, two sample t test.

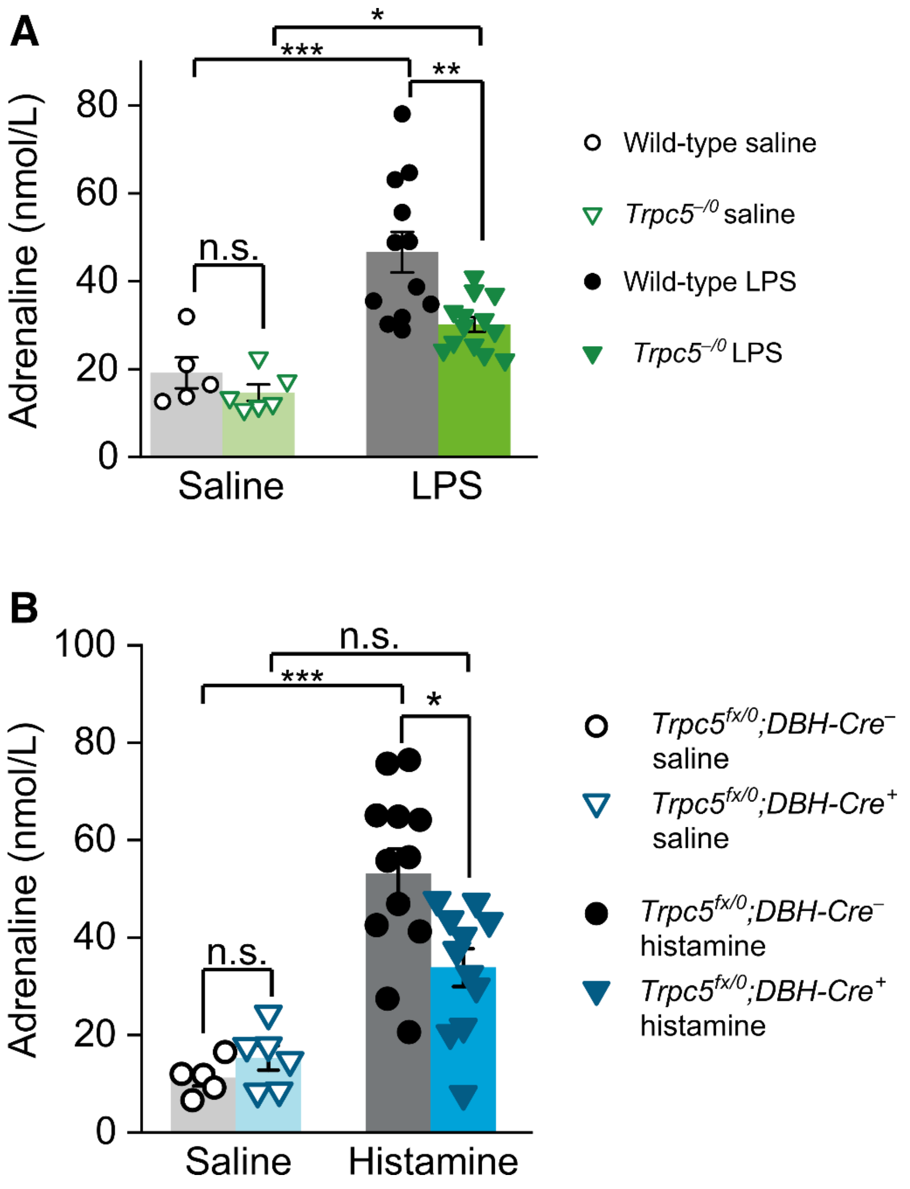

Appendix Figure S2: Stress-induced adrenaline secretion in *Trpc5-deficient* mice.

(A) Plasma adrenaline levels 60 min after administration of LPS in WT and *Trpc5*$^{-/0}$ mice. Mean ± s.e.m. (*Trpc5*$^{-/0}$: p = 0.0336, n = 6 for LPS and n = 13 for saline, wild-type: p = $1.72×10^{-4}$, n = 12 for LPS and n = 5 for saline). Two-way ANOVA p = $2.03×10^{-6}$. Post hoc means comparison with Bonferroni correction p = $6.93×10^{-4}$ for genotype and p = $3.67×10^{-6}$ for treatment; between genotypes, LPS treatment p = 0.00293, saline treatment p = 1. **(B)** Plasma adrenaline values 30 min after histamine or saline administration in a histamine tolerance test (*Trpc5*$^{fx/0}$*;DBH-Cre*$^{-}$:p = $5.46×10^{-4}$, n = 12 for histamine and n = 5 for saline, *Trpc5*$^{fx/0}$*;DBH-Cre*$^{+}$: p = 0.464, n = 11 for histamine and n = 6 for saline). Mean ± s.e.m., two-way ANOVA p = $7.45×10^{-5}$. Post hoc means comparison with Bonferroni correction p = 0.0077 for genotype and p = $7.85×10^{-5}$ for treatment; between genotypes, histamine treatment p = 0.0182, saline treatment p = 1 n.s.: p > 0.05; * p ≤0.05; **: p ≤ 0.01; ***: p ≤ 0.001).

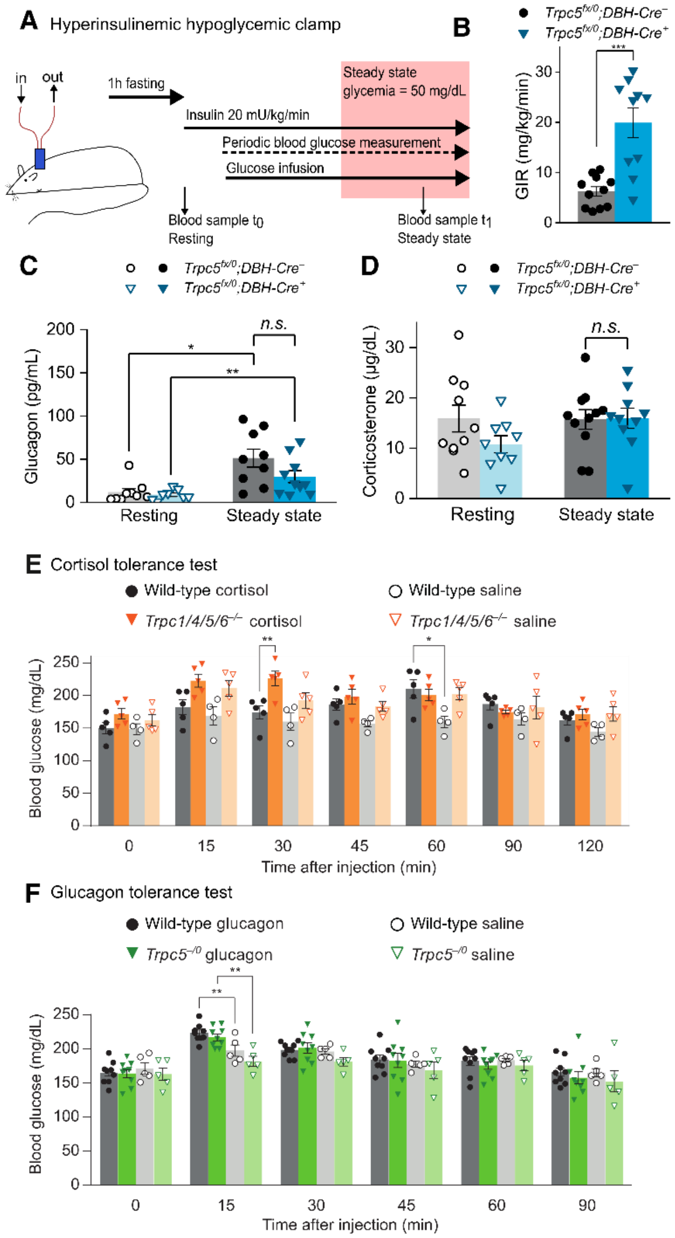

Appendix Figure S3: The interplay between glucagon and cortisol is not altered in TRPC5 deficient mice.

(A) Schematic representation of the hyperinsulinemic hypoglycemic clamp experiments with indication of the timepoints of blood sampling. The first sample, t0, was taken before the insulin infusion started, the second sample was taken during steady state 50 mg/dL blood glucose levels. **(B)** The glucose infusion rate during steady state conditions, p = 1.77×10^{-4}. **(C)** The plasma glucagon concentration before and during hypoglycemia, mean ± s.e.m., pairwise t-test *Trpc5*$^{fx/0}$;*DBH-Cre*$^{+}$, p = 0.00715, *Trpc5*$^{fx/0}$;*DBH-Cre*$^{-}$, p = 0.0122; steady state p = 0.0976. **(D)** The plasma corticosterone levels before and during hypoglycemia (50 mg/dL blood glucose) (B, C, D) n = 8-11 per group. **(E)** Glucagon- and cortisol-evoked blood glucose rise is not reduced by TRPC inactivation. Cortisol tolerance test: Time course of the blood glucose levels after the injection of cortisol for *Trpc1/4/5/6*$^{-/-}$ mice. (*Trpc1/4/5/6*$^{-/-}$: n = 5 for cortisol, n = 5 for saline; wild-type: n = 5 for cortisol, n = 4 for saline). Cortisol, 30 min p = 0.00964; wild-type, 60 min p = 0.0226. **(F)** Glucagon tolerance test: Time course of the blood glucose levels after the application of glucagon. Mice which received saline intraperitoneally serve as a reference. *Trpc5*$^{-/0}$: n = 9 for glucagon, n = 5 for saline; wild-type: n = 9 for glucagon, n = 5 for saline; 15 min; wild-type p = 0.00916, *Trpc5*$^{-/0}$ p = 0.00123. (B, D-F) Mean ± s.e.m., two sample t-test, n.s. p > 0.05, * p < 0.05, ** p< 0.01, *** p< 0.001.

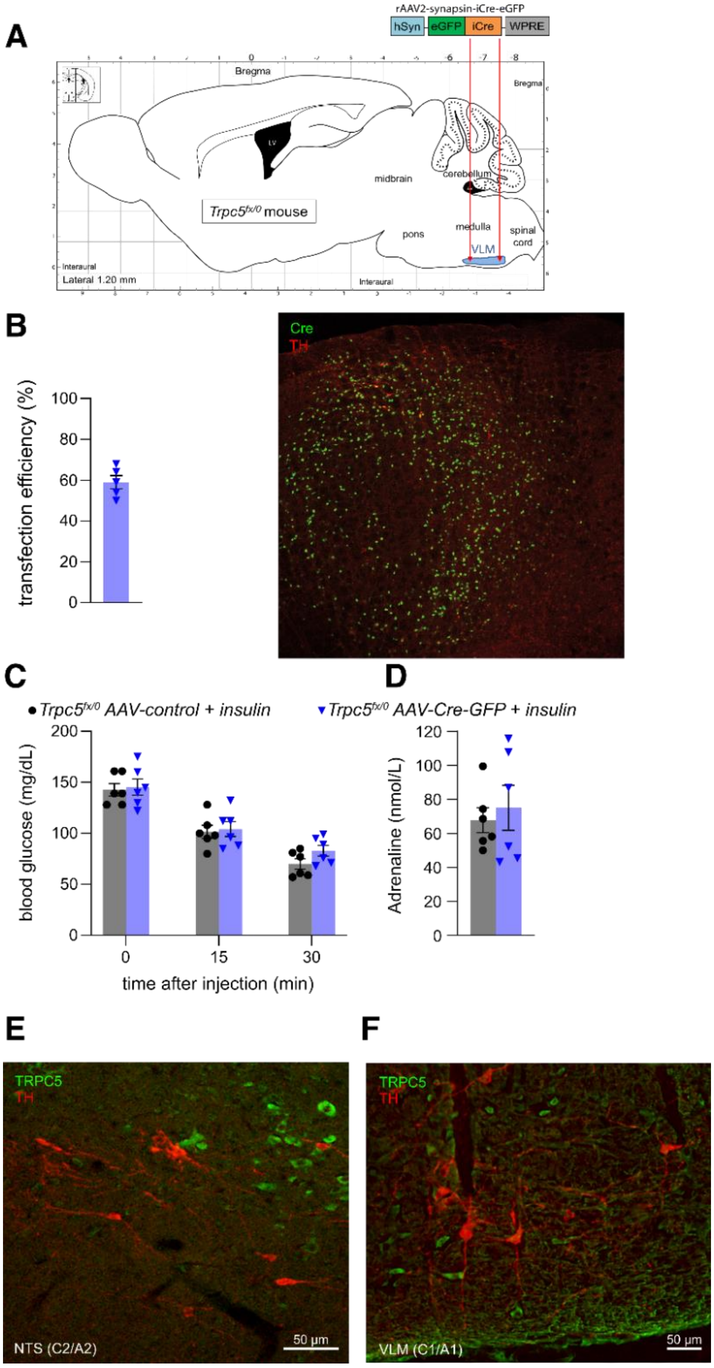

Appendix Figure S4: Analysis of the role of TRPC5 in catecholaminergic neurons of the VLM and NTS.

(A) Scheme for the bilateral injections of AAV2-Cre-GFP into the VLM of *Trpc5*$^{fx/0}$ mice. **(B)** left: Transduction rate in catecholaminergic RVLM neurons. right: Representative immunohistology of coronal brain sections, using anti-TH antibody (red) in *Trpc5*$^{fx/0}$ mice injected with AAV2-Cre-GFP, n = 5 independent transductions. **(C, D)** ITT. (C) Time course of the blood glucose levels after insulin application for *Trpc5*$^{fx/0}$ mice injected with AAV2-Cre-GFP or AAV2-GFP as control. (D) Corresponding plasma adrenaline levels. (*Trpc5*$^{fx/0}$ + AAV2-Cre-GFP: n = 6; *Trpc5*$^{fx/0}$ + AAV2-GFP: n = 6) **(E)** Representative image of TH (red) immunoreactive cells of the C2/A2 area (NTS) at approximately Bregma -7.5 mm in *Trpc5*$^{+/0}$ male mice (n = 3). **(F)** Representative image of TH immunoreactive cells (red) of the C1/A1 area (VLM) at approximately Bregma -6.5 mm in *Trpc5*$^{+/0}$ male mice (n = 3). The merged images shown in E and F, indicate that TRPC5 protein (green) was not co-expressed by the TH (red) immunoreactive cells of either the NTS (98 %; 1 out of 55 TH+ cells) or VLM (97 %; 2 out of 60 TH+ cells).

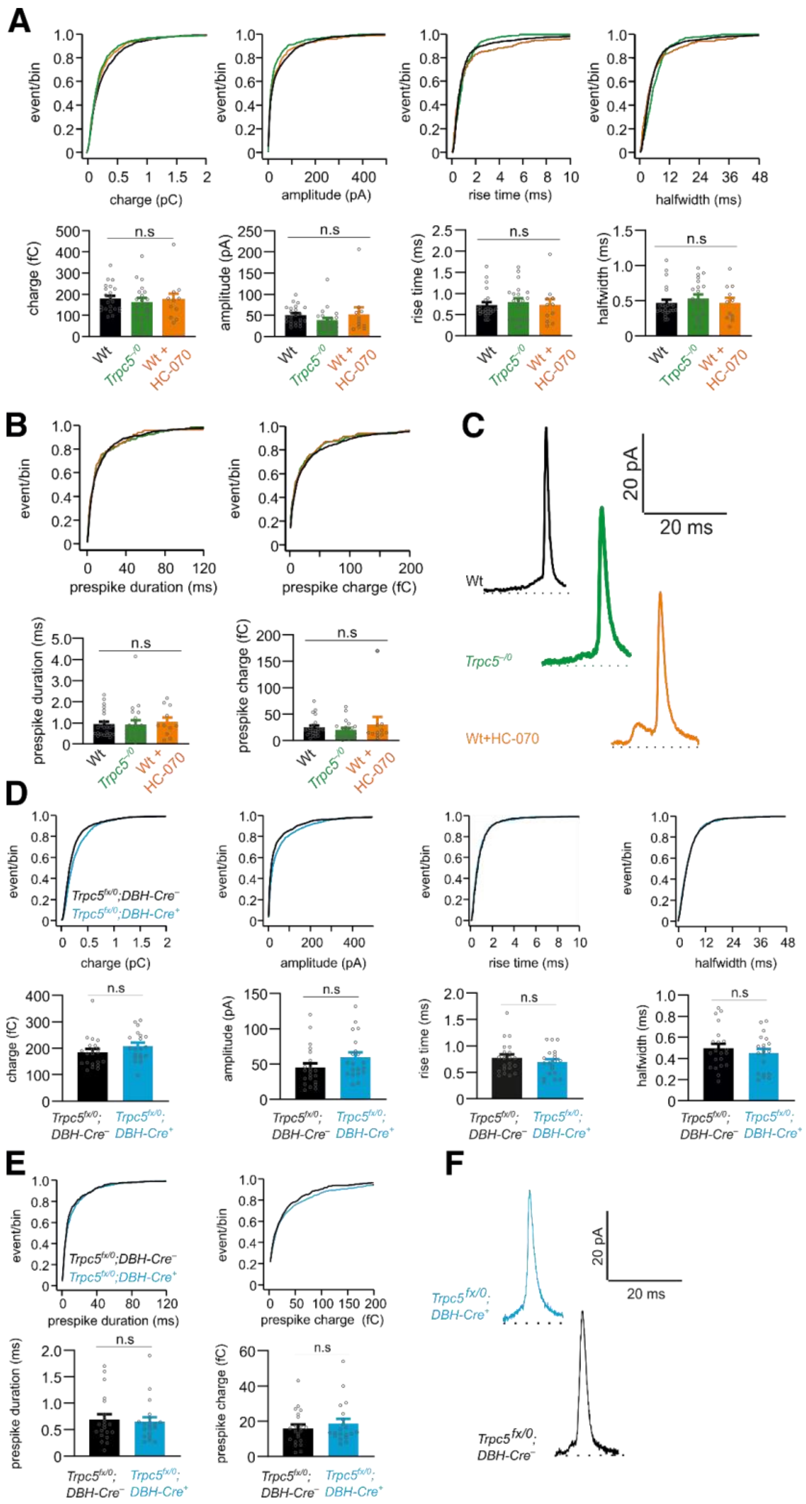

Appendix Figure S5: Properties of the amperometric release events are not affected by TRPC5 activity.

(A) Properties of amperometric events during the spike phase and the **(B)** pre-spike phase displayed as (top row) cumulative frequency distribution and (bottom row) cell-weighted averages for wild-type, and wild-type with the HC-070 Trpc5 inhibitor. Note that neither the spike phase nor pre-spike phase parameters are changed in the absence of TRPC5 or its inhibition (HC-070). (A-B) Data were collected from wt, n = 23, *Trpc5*$^{-/0}$, n = 21, wt+HC070, n = 11. One-way ANOVA, Tukey Kramer test. **(C)** Exemplary single release events illustrating the unchanged quantal release in either *Trpc5*-deficient or HC070 treated wt chromaffin cells when compared to wt controls. **(D-F)** as B-D but recorded in cells isolated from *Trpc5*$^{fx/0}$*;DBH-Cre*$^{-}$ (n = 20) and *Trpc5*$^{fx/0}$*;DBH-Cre*$^{+}$ mice (n = 21). Mann Whitney U test. Bar graphs are displayed as mean ± s.e.m..

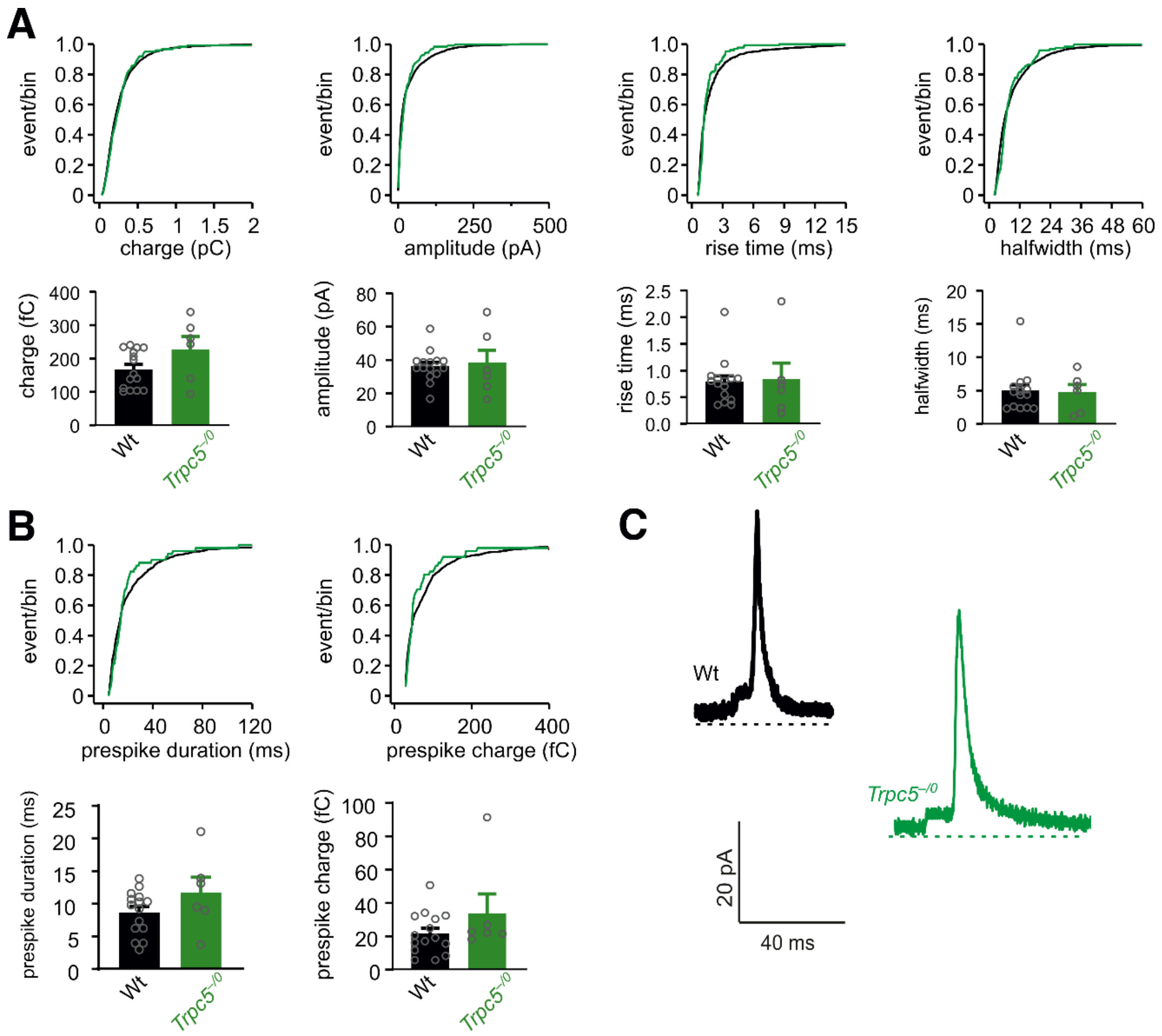

Appendix Figure S6: Genetic loss of TRPC5 does not alter quantal size of catecholamine secretion in response to muscarine stimulation.

(**A**) Properties of amperometric events during the spike phase and prespike phase (**B**) displayed as cumulative frequency distribution (top rows) and cell-weighted averages (bottom rows) for the indicated groups (numbers indicate averaged cells). Note that neither the spike phase nor prespike phase parameters are changed in the absence of TRPC5. (A-B) Data were collected from wt, n = 15 and *Trpc5*$^{-/0}$ n = 6 cells. Mann Whitney U test. (**C**) Exemplary single release events for the indicated groups illustrate the unchanged quantal release. Bar graphs are displayed as mean ± s.e.m..

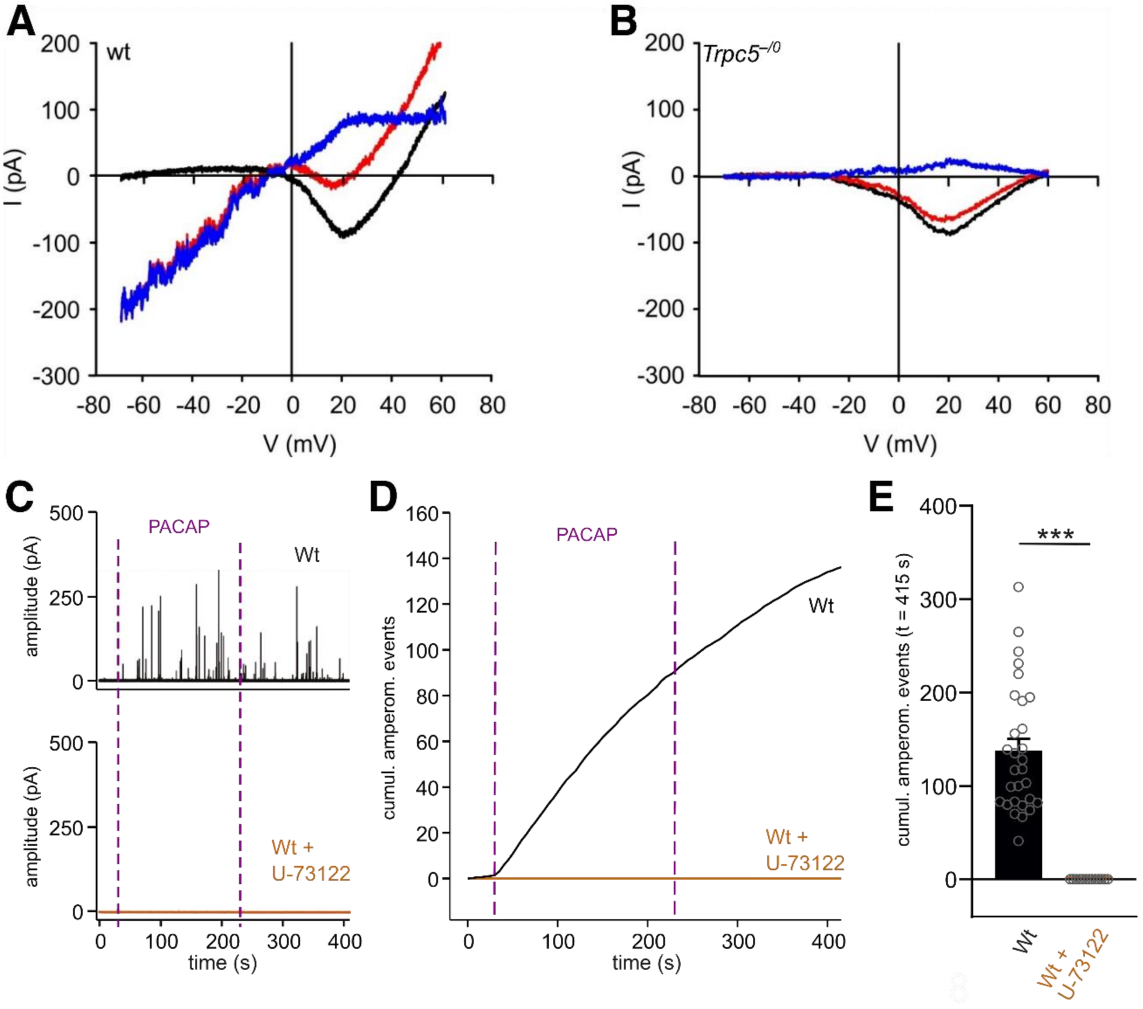

Appendix Figure S7: PACAP-activated cation currents and phospholipase C dependent catecholamine secretion in chromaffin cells from wt and *Trpc5*$^{-/0}$ mice. (A,B) Exemplary current-voltage relationship (IV) of Wt (**A**) and *Trpc5*$^{-/0}$ chromaffin cells (**B**) before (black) and during (red) PACAP-induced burst-like current activity (voltage ramp from -70 to +60 mV, 400 ms duration). wt cells show bell-shaped Ca^{2+}-current activity under baseline conditions (black) and respond with additional strong inward and outward currents during PACAP-application (red). PACAP-induced currents (blue) of wt cells exhibit a reversal potential (V_{rev}) around -11 mV. PACAP-induced inward and outward currents depend on TRPC5 channel activity, as no comparable currents were detected in *Trpc5*$^{-/0}$ cells. **(C)** Exemplary amperometric recordings in response to PACAP application for wt and wt cells preincubated with PLC blocker U-73122 (10 µM). **(D)** Mean cumulative plot of amperometric events for the indicated groups. Dashed purple lines indicate the beginning and the end of PACAP application (1 µM, 200 seconds). **(E)** Total amperometric events after 415 seconds for the indicated groups wt, n = 29; wt+U-73122, n = 13. Mann-Whitney U test (*** p<0.001), bar graphs are displaying mean ± s.e.m..

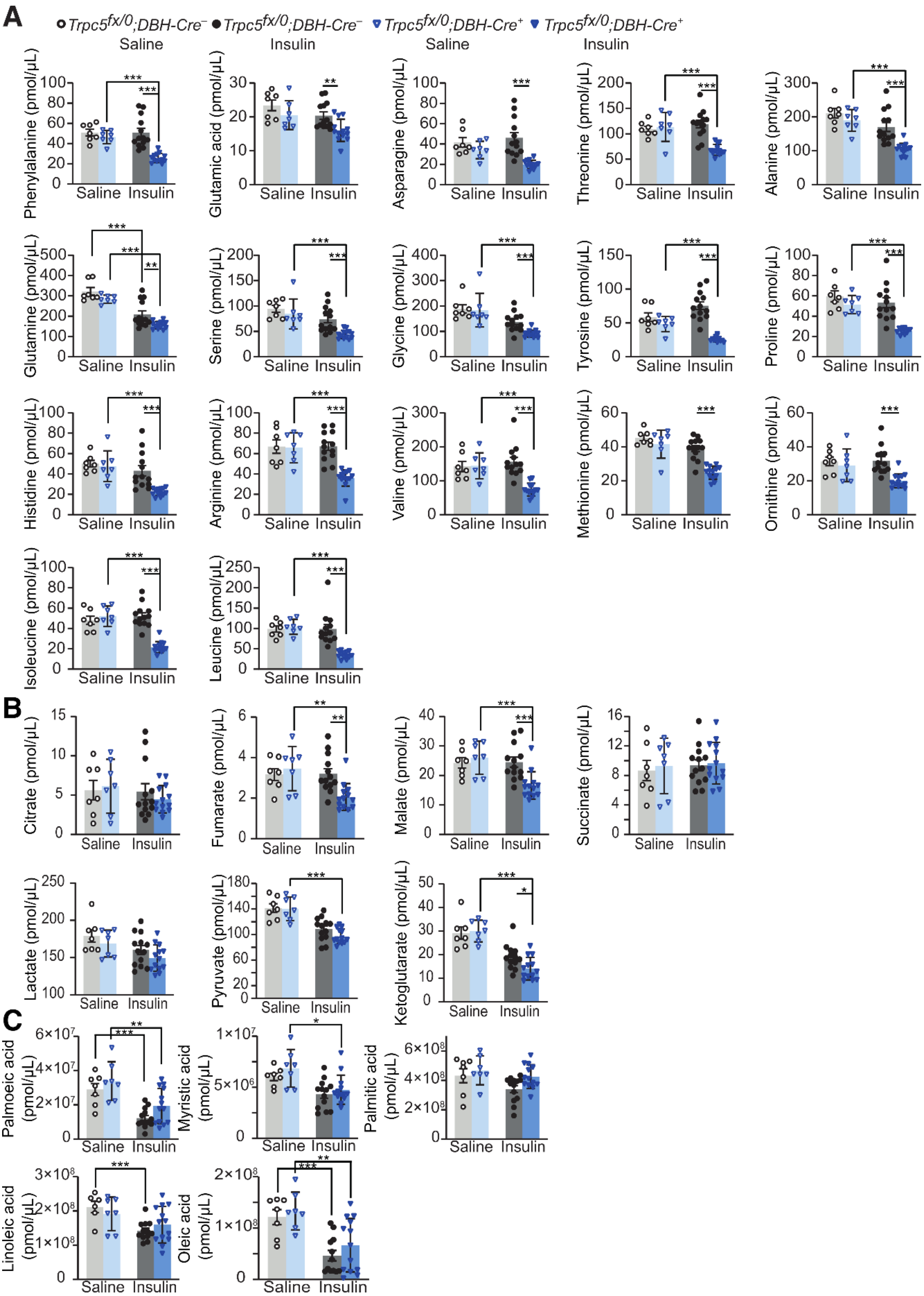

A
Trpc5fx/0;DBH-Cre⁻ Saline
Trpc5fx/0;DBH-Cre⁻ Insulin
Trpc5fx/0;DBH-Cre⁺ Saline
Trpc5fx/0;DBH-Cre⁺ Insulin
Phenylalanine (pmol/μL)
Glutamic acid (pmol/μL)
Asparagine (pmol/μL)
Threonine (pmol/μL)
Alanine (pmol/μL)
Glutamine (pmol/μL)
Serine (pmol/μL)
Glycine (pmol/μL)
Tyrosine (pmol/μL)
Proline (pmol/μL)
Histidine (pmol/μL)
Arginine (pmol/μL)
Valine (pmol/μL)
Methionine (pmol/μL)
Ornithine (pmol/μL)
Isoleucine (pmol/μL)
Leucine (pmol/μL)
B
Citrate (pmol/μL)
Fumarate (pmol/μL)
Malate (pmol/μL)
Succinate (pmol/μL)
Lactate (pmol/μL)
Pyruvate (pmol/μL)
Ketoglutarate (pmol/μL)
C
Palmoeic acid (pmol/μL)
Myristic acid (pmol/μL)
Palmitic acid (pmol/μL)
Linoleic acid (pmol/μL)
Oleic acid (pmol/μL)
Saline
Insulin

Appendix Figure S8: Alterations in plasma amino acids, TCA cycle metabolites and fatty acids in *Trpc5$^{fx/0}$;DBH-Cre^{+}* and *Trpc5$^{fx/0}$;DBH-Cre$^{–}$* mice under insulin evoked hypoglycemia

(A – C) Plasma levels of (A) amino acids, (B) TCA cycle compounds, and (C) fatty acids 30 min after the injection of saline (basal) or insulin (HG; 2.25 U/kg insulin, i.p.) for a catecholamine-specific *Trpc5* KO. *Trpc5$^{fx/0}$;DBH-Cre^{+}*: n = 15 for insulin, n = 7 for saline; *Trpc5$^{fx/0}$;DBH-Cre$^{–}$*: n = 13 for insulin, n = 7 for saline. Phenylalanine, *Trpc5$^{fx/0}$;DBH-Cre^{+}* p = 1.42×10^{-7}, insulin p = 6.69×10^{-6}; Glutamic acid, insulin, p = 0.00526; asparagine, insulin p = 5.72×10^{-5}; Threonine, *Trpc5$^{fx/0}$;DBH-Cre^{+}* p = 5.96×10^{-5}, insulin p = 1.63×10^{-6}; Alanine, *Trpc5$^{fx/0}$;DBH-Cre^{+}* p = 3.77×10^{-7}, insulin p = 0.000108; Glutamine, *Trpc5$^{fx/0}$;DBH-Cre^{+}* p = 2.14×10^{-11}, *Trpc5$^{fx/0}$;DBH-Cre$^{–}$* p = 0.000670, insulin p = 0.00814; Serine, *Trpc5$^{fx/0}$;DBH-Cre^{+}* p = 0.000170, insulin p = 0.000489; Glycine, *Trpc5$^{fx/0}$;DBH-Cre^{+}* p = 0.000104, insulin p = 0.000243; Tyrosine, *Trpc5$^{fx/0}$;DBH-Cre^{+}* p = 2.91×10^{-6}, insulin p = 1.32×10^{-8}; Proline, *Trpc5$^{fx/0}$;DBH-Cre^{+}* p = 9.16×10^{-9}, insulin p = 7.77×10^{-6}; Histidine, *Trpc5$^{fx/0}$;DBH-Cre^{+}* p = 1.46×10^{-5}, insulin p = 0.000404; Arginine, *Trpc5$^{fx/0}$;DBH-Cre^{+}* p = 1.43×10^{-5}, insulin p = 5.00×10^{-7}; Valine *Trpc5$^{fx/0}$;DBH-Cre^{+}* p = 2.81×10^{-5}, insulin p = 3.60×10^{-6}; Methionine, insulin p = 3.66×10^{-7}; Ornithine, insulin, p = 7.25×10^{-5}; Isoleucine, *Trpc5$^{fx/0}$;DBH-Cre^{+}* p = 5.69×10^{-8}, insulin p = 4.37×10^{-9}; Leucine, *Trpc5$^{fx/0}$;DBH-Cre^{+}* p = 9.94×10^{-10}, insulin p = 7.97×10^{-6}; Fumarate, *Trpc5$^{fx/0}$;DBH-Cre^{+}* p = 0.00202, insulin p = 0.00130; Malate, *Trpc5$^{fx/0}$;DBH-Cre^{+}* p = 0.0000760, insulin p = 0.000986; Pyruvate, *Trpc5$^{fx/0}$;DBH-Cre^{+}* p = 5.50×10^{-6}; Ketoglutarate, *Trpc5$^{fx/0}$;DBH-Cre^{+}* p = 1.03×10^{-6}, insulin p = 0.0189; Palmoeic acid, *Trpc5$^{fx/0}$;DBH-Cre^{+}* p = 0.00895, *Trpc5$^{fx/0}$;DBH-Cre$^{–}$* p = 7.94×10^{-5}; Myristic acid, *Trpc5$^{fx/0}$;DBH-Cre^{+}* p = 0.0110; Linoleic acid, *Trpc5$^{fx/0}$;DBH-Cre$^{–}$* p = 0.000524; Oleic acid, *Trpc5$^{fx/0}$;DBH-Cre^{+}* p = 0.00767, *Trpc5$^{fx/0}$;DBH-Cre$^{–}$* p = 0.000495. HG, hypoglycemia; TCA, tricarboxylic acid cycle. (A-C) *: p<0.05, **: p<0.01, ***: p<0.001, two sample t test.

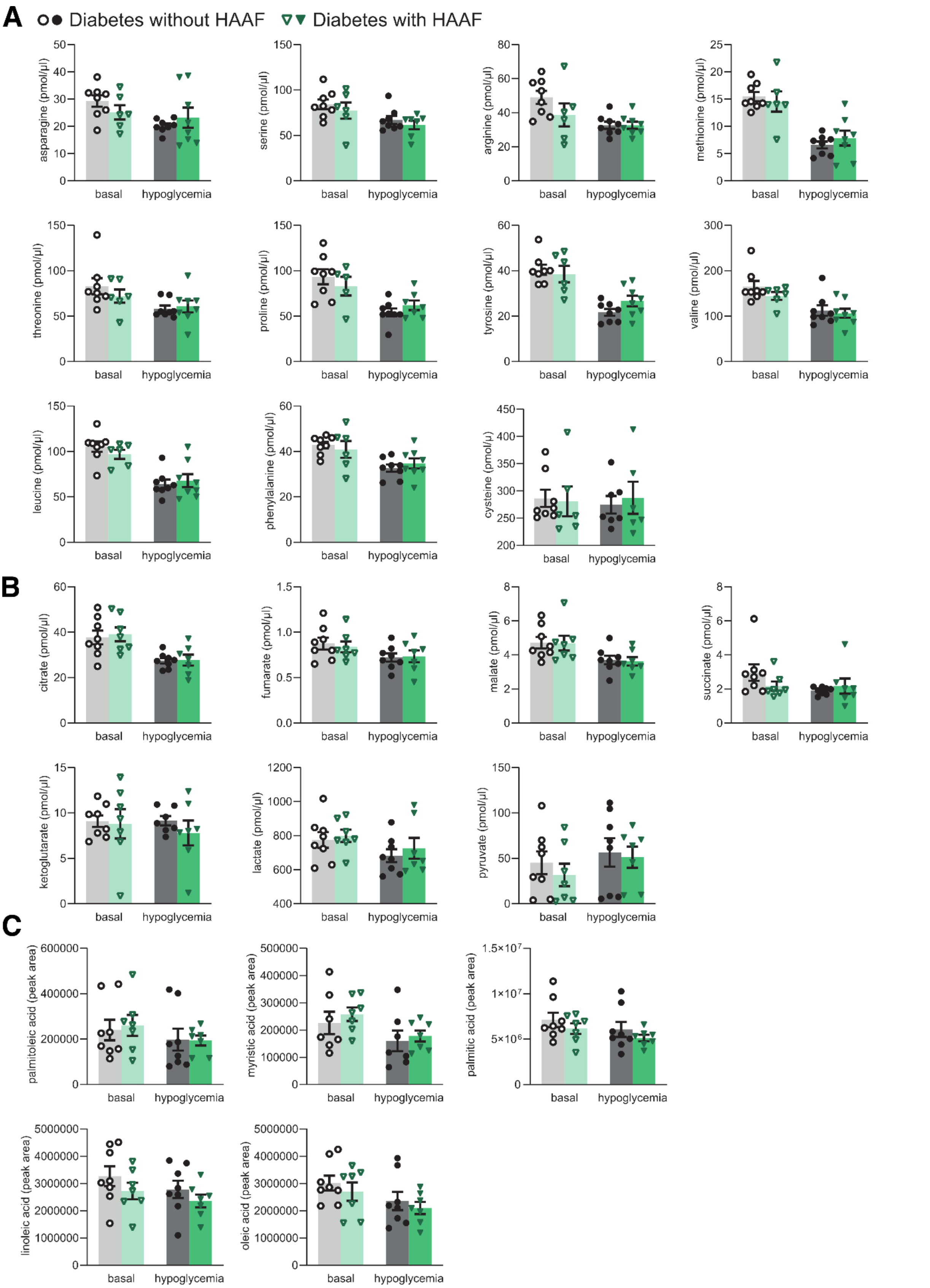

Appendix Figure S9: Analysis of plasma amino acids, TCA cycle metabolites and fatty acids in HAAF patients under insulin evoked hypoglycemia

(A – C) Plasma levels of (A) amino acids, (B) TCA cycle compounds, and (C) fatty acids for diabetic humans diagnosed with HAAF before (basal) and during controlled hypoglycemia (HG; 60-70 mg/dL). (n = 7 for HAAF patients, n = 8 for diabetes patients without HAAF) HG, hypoglycemia.

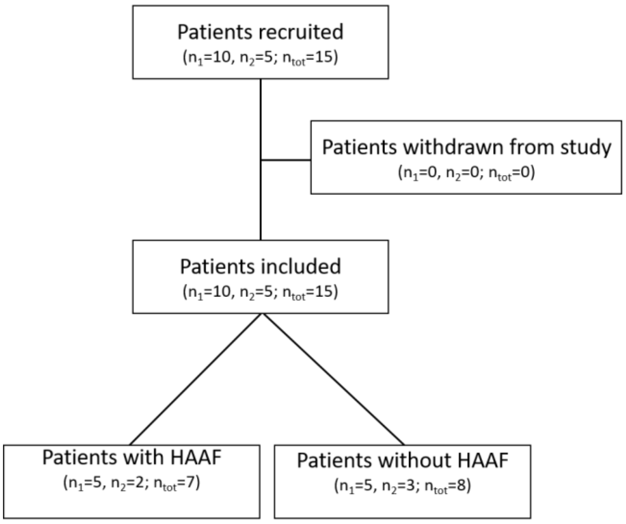

Inclusion criteria:
Age between 18 and 75 years
BMI between 20 and 35 kg/m2
Type 1 diabetes diagnosis

Exclusion criteria:
Secondary types of diabetes
Current pregnancy
Acute infection/fever
Immune-suppressant therapy
Severe psychiatric diseases requiring treatment
Known alcohol or drug dependency
Severe heart-, kidney-, or liver-insufficiency
Cancer or other malignant diseases within the last 5 years
Infectious diseases (hepatitis B, C, E, HIV)
Severe autoimmune diseases
Participation in other studies
Anemia or disorders of bone marrow

Appendix Figure S10: Flowchart, inclusion and exclusion criteria for the patients recruited in the study. The initial study design and approval was obtained for 10 patients in total, 5 in each group, reflected by n_1 in the flowchart. An ethical approval for the recruitment of more patients was requested and approved, and additional patients were recruited in the study (n_2). The total number of patients in each group, included in the analysis in this manuscript is indicated with n_{tot}.
