# Supplementary figures and images for: TRPC5 controls the adrenaline-mediated counter regulation of hypoglycemia
Source: EMBO J. 2024 Oct 7;43(23):5813–36. doi: 10.1038/s44318-024-00231-0 (PMC11612138; doi:10.1038/s44318-024-00231-0)

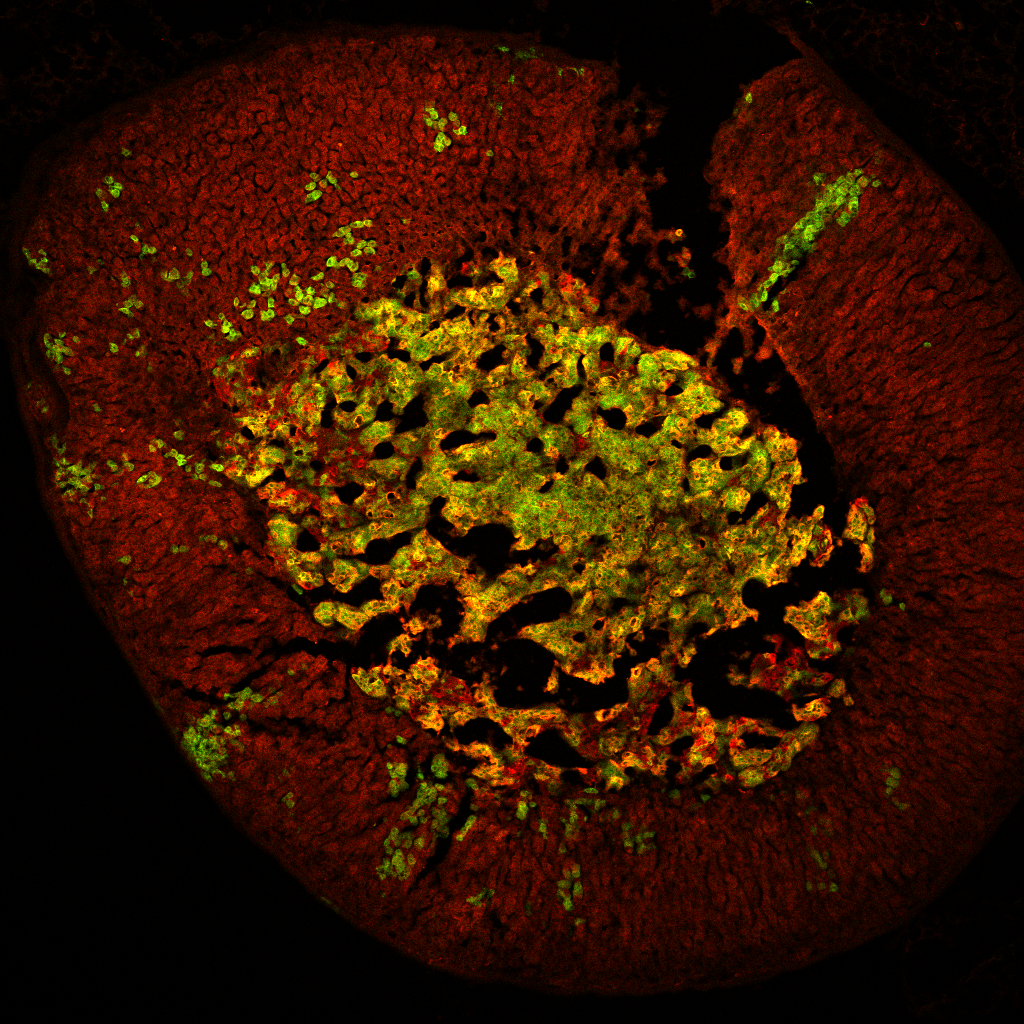

Supplement: Supplementary file 7 — Source data Fig. 4 [file 44318_2024_231_MOESM7_ESM.zip › Figure 4/4G/4G MERGE.tif]

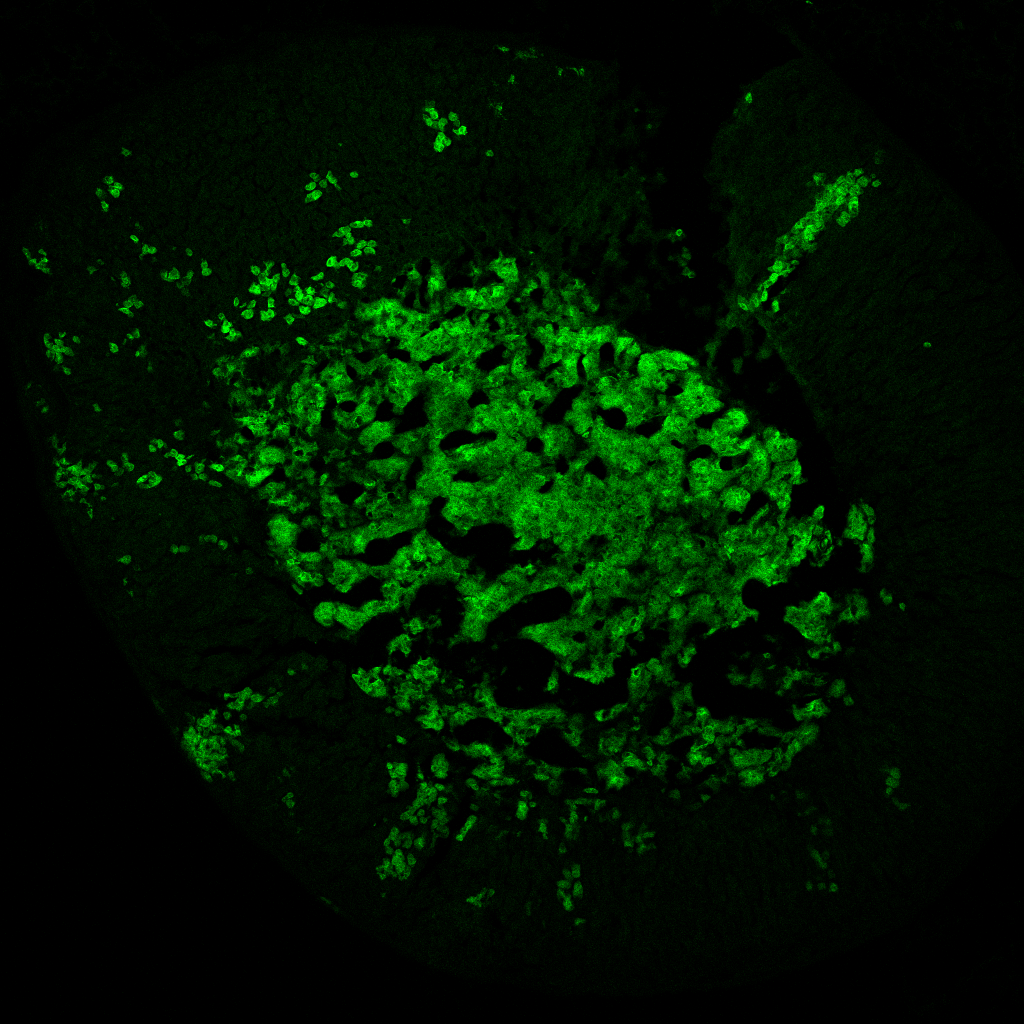

Supplement: Supplementary file 7 — Source data Fig. 4 [file 44318_2024_231_MOESM7_ESM.zip › Figure 4/4G/4G GREEN.tif]

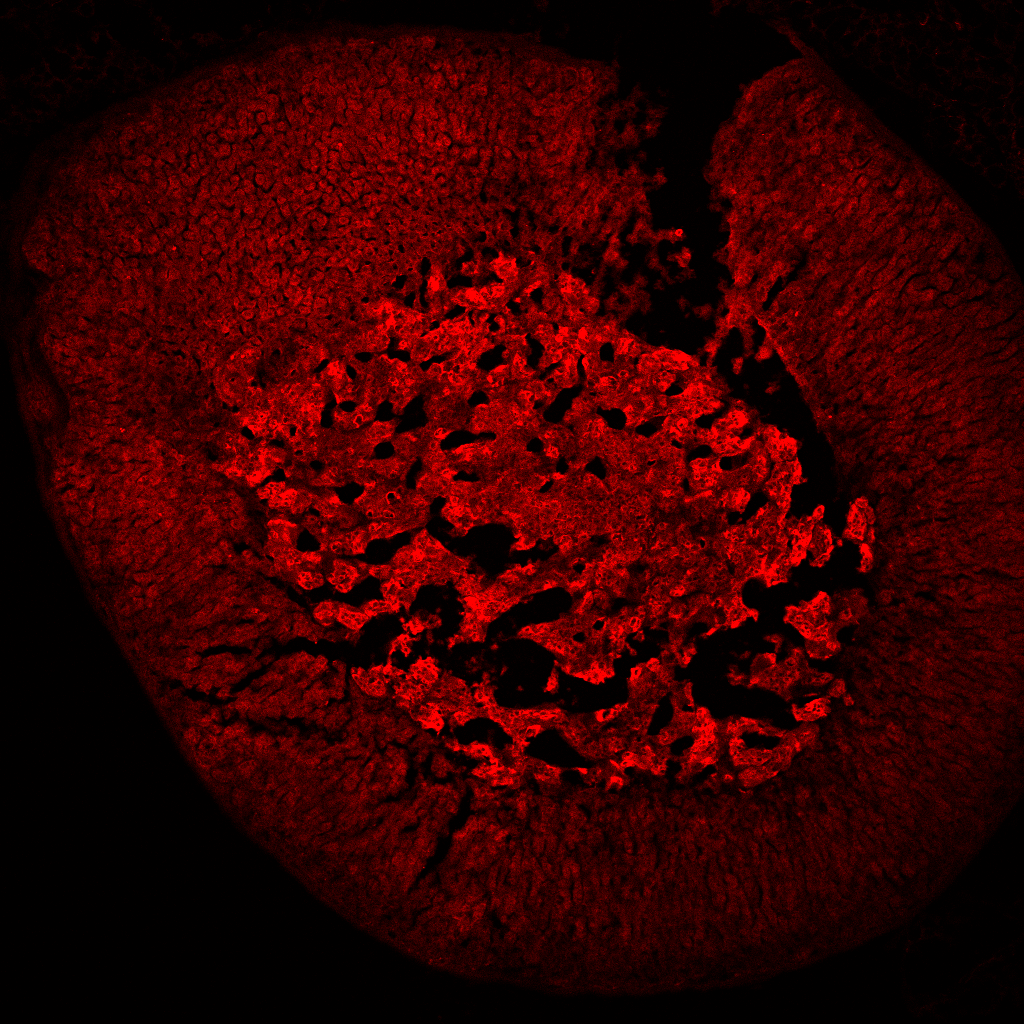

Supplement: Supplementary file 7 — Source data Fig. 4 [file 44318_2024_231_MOESM7_ESM.zip › Figure 4/4G/4G RED.tif]
